# Teamwork Training With a Multiplayer Game in Health Care: Content Analysis of the Teamwork Principles Applied

**DOI:** 10.2196/38009

**Published:** 2022-12-09

**Authors:** Lara van Peppen, Tjitske J E Faber, Vicki Erasmus, Mary E W Dankbaar

**Affiliations:** 1 Institute of Medical Education Research Rotterdam Erasmus University Medical Center Rotterdam Netherlands; 2 Learning and Innovation Center Avans University of Applied Sciences Breda Netherlands; 3 Anesthesiologie, Pijn en Palliatieve Geneeskunde Radboud Universitair Medisch Centrum Nijmegen Netherlands; 4 Implementation Research and Interprofessional Education Erasmus University Medical Center Rotterdam Netherlands

**Keywords:** teamwork, skills training, serious games, multiplayer game, medical students, content-analysis, health care, interprofessional teamwork

## Abstract

**Background:**

In health care, teamwork skills are critical for patient safety; therefore, great emphasis is placed on training these skills. Given that training is increasingly designed in a blended way, serious games may offer an efficient method of preparing face-to-face simulation training of these procedural skills.

**Objective:**

This study aimed to investigate the teamwork principles that were used during gameplay by medical students and teamwork experts. Findings can improve our understanding of the potential of serious games for training these complex skills.

**Methods:**

We investigated a web-based multiplayer game designed for training students’ interprofessional teamwork skills. During gameplay, 4 players in different roles (physician, nurse, medical student, and student nurse) had to share information, prioritize tasks, and decide on next steps to take in web-based patient scenarios, using one-to-one and team chats. We performed a qualitative study (content analysis) on these chats with 144 fifth-year medical students and 24 health care teamwork experts (as a benchmark study) playing the game in groups of 4. Game chat data from 2 scenarios were analyzed. For the analysis, a deductive approach was used, starting with a conceptual framework based on Crew Resource Management principles, including shared situational awareness, decision-making, communication, team management, and debriefing.

**Results:**

Results showed that most teamwork principles were used during gameplay: shared situational awareness, decision-making (eg, re-evaluation), communication (eg, closed loop), and team management (eg, distributing the workload). Among students, these principles were often used on a basic level. Among experts, teamwork principles were used with more open forms of speak up and more justification of decisions. Some specific Crew Resource Management principles were less observed among both groups, for example, prevention of fixation errors and use of cognitive aids. Both groups showed relatively superficial debriefing reflections.

**Conclusions:**

Playing a multiplayer game for interprofessional teamwork appears to facilitate the application of teamwork principles by students in all important teamwork domains on a basic level. Expert players applied similar teamwork principles on a moderately high complexity level. Some teamwork principles were less observed among both students and expert groups, probably owing to the artifacts of the game environment (eg, chatting instead of talking). A multiplayer game for teamwork training can elicit the application of important, basic teamwork principles, both among novices and experts, and provides them with a flexible, accessible, and engaging learning environment. This may create time for exercising more complex skills during face-to-face training.

## Introduction

### Background

Across the globe, in high-stakes environments (such as aviation, nuclear power industries, and health care), teams work together to prevent harm. A team may be defined as “two or more persons with a common goal that requires interdependence and adaptive functioning” [1]. Teams may exist offline and on the web, including groups of individuals in different locations brought together by technology to accomplish a common goal or task [2]. For the team to achieve its goals, team members must perform both taskwork (meaning specific work-related activities) and teamwork, including sharing knowledge, coordinating behaviors, and trusting one another [3]. Studies in a variety of fields have established the benefits of both teamwork in itself [4] and training in team-based skills such as interdependent collaboration, communication, and shared decision-making [5,6].

In health care, many adverse events are associated with systematic issues and human error [7], such as problems in communication and lack of leadership [8,9]. One of the challenges in medical practice is that health care professionals perform interdependent tasks, with different roles and responsibilities, sharing the common goals of quality and safety in care. Especially in complex and acute emergency situations, environmental and patient conditions can easily alter judgments and affect both individual and team decision-making [10]. To successfully navigate such situations, good communication and cooperation are essential. However, in such situations, increased stress levels can disrupt good communication and cooperation. It becomes more challenging for team members to extract relevant information from other team members and individuals, communicate this information, and make joint decisions. Providing good and safe patient care requires more than medical knowledge and technical skills. Given the interdisciplinary nature of the work and the necessity for cooperation among health care professionals, teamwork is equally essential [9,11]. To reduce risks in medical practice, health care professionals should be equipped with the skills needed to effectively operate in dynamic safety-critical situations [12]. In other words, they require the skills needed to build and maintain adequate *situational awareness* in complex, dynamic, and high-risk situations. Therefore, training medical students in teamwork has received increased attention in the past decade, and teamwork principles are being introduced into several medical school curricula [6,13-17].

Team training encompasses a broad range of strategies, most commonly targeting communication, situational awareness, leadership, role clarity, and coordination [6]. In health care, the most commonly used type of training is Crew Resource Management (CRM) [18-20]. CRM is adopted from studies on aviation teams [21] and is currently used in many high-risk domains to train professionals. CRM includes team training, simulation, and interactive group debriefings. The main purpose of CRM is to teach professionals about the limitations of human performance and foster their understanding of cognitive errors and impact of stressors on decision-making processes. Teamwork skills being trained following CRM principles include, for instance, communicating, exerting leadership, anticipating and planning, managing workload distribution, and re-evaluating actions [22]. It is important to note that a shared framework for all CRM principles is missing [19] and that there is no universal CRM training program—organizations customize their CRM training to best suit their individual needs.

### Training of Teamwork Skills

Teamwork skills are generally taught face-to-face, with a group in a simulation setting. Simulation-based education allows students to acquire new knowledge and skills in an environment that more or less resembles the future work situation and allows for live interaction between the team members. However, such training is cost intensive. To use time and resources more efficiently, many education systems shifted away from traditional face-to-face classes to blended learning—a combination of web-based instruction and face-to-face classes [23]. In a blended design, web-based learning is often implemented before face-to-face classes to create knowledge and skills on a basic level; face-to-face time is used to train skills on a high level and provide and process feedback [24]. Implementing blended learning can bring about a shift toward a more active approach to learning that focuses on the individual student [25,26]. Students have more control over their learning process and can adapt learning activities to their own needs and preferences [27]. For example, they can work at their own pace, relearn specific parts of the material, and choose when to learn. Numerous studies have shown that blended learning is at least as effective for the acquisition of knowledge and skills compared with traditional learning and that students are equally motivated to learn [24,28-32].

Web-based learning environments (such as e-learning modules or simulation programs) have proven to be valuable tools for developing and refining skills [29,33]. Web-based simulation training allows students to acquire new knowledge and skills in an environment that more or less resembles their future work situation. The use of web-based patients (ie, computerized clinical case simulations) as a learning tool is associated with enhanced cognitive clinical skills [34]. In addition, the use of technology-enhanced simulation training is consistently associated with large effects on learning outcomes for knowledge, skills, and behavior [35]. Group interactions play a major role in the development of teamwork skills. A promising web-based environment for the training of teamwork skills is a multiplayer serious game. Serious games can offer challenging and realistic scenarios that encourage learners to explore various situations and perform activities as a team, thus allowing mutual interaction [36]. Moreover, games can provide immediate feedback about the user’s actions and guidance in learning, which is mainly important for novices [37]. Through serious games, learners can experience the consequences of their actions in a safe and controlled environment.

In summary, well-designed multiplayer serious games have the potential to enhance students’ teamwork skills, especially in a blended training design, at a fraction of the cost of full-scale simulations. In a blended design, skills are pretrained in a web-based setting, allowing an activating, efficient, and flexible form of learning.

### This Study

The interest in serious games has grown significantly over the past decades, especially in anesthesiology and obstetrics [38], and serious games are increasingly being implemented in health education for a variety of users [39]. Despite its potential, studies in the field of serious games targeting cognitive (clinical) skills are still sparse. A simulation game designed to teach residents (cognitive) acute care skills, as a preparation for face-to-face training, has been shown to be effective in improving these skills [40]. In addition, a web-based emergency room game for interprofessional education improved teamwork attitudes among medical and nursing students [41]. Moreover, Keith et al [42] found that playing video games as a team improved team performance, probably related to team cohesion processes. However, robust studies on game effectiveness and design choices are still inconclusive owing to the low quality of the evidence and paucity of studies included in meta-analyses [43-45].

Given the increasing relevance of blended training designs, it is important to gain insight into the potential of web-based learning environments for the development of teamwork skills.

We know that to learn a skill, tasks must be applied in an authentic context [37]; consequently, a game environment should stimulate the application of that specific skill. It is not yet clear which teamwork skills can be facilitated by serious games (as part of a blended training design). Therefore, this study examined the extent to which teamwork skills are used in the multiplayer game, *Team Up!* (more information about this game is provided in the *Materials* subsection of the *Methods* section). Findings can contribute to a better understanding of the potential of serious games for developing teamwork skills. In addition, they can inform designers and trainers in implementing an effective blended design, by helping to decide what skills require more attention in the face-to-face training sessions.

This study aimed to investigate the teamwork principles that were used in the game. As teamwork and communication between team members in the game occurred through individual and team chat, we qualitatively analyzed the game chat data. The type of teamwork principles used may relate to the level of teamwork experience of the players. Therefore, we performed *2 qualitative studies*. The first (main) study was conducted with fifth-year medical students (the primary target group of the game) and the second (conceptual replication) study was conducted with teamwork experts. The second study was conducted to increase the robustness and ecological validity of our findings. Teamwork experts were CRM trainers or physicians and residents in anesthesia department, both well trained in teamwork and use of CRM principles. We hypothesized that both groups of participants would apply the main teamwork principles during gameplay.

## Methods

### Ethics Approval

The study was approved by the Medical Ethics Review Committee for medical research using human participants from the relevant medical university (protocol ID BD1).

### Recruitment and Data Collection

#### Study 1

The game, *Team Up!* was played during a mandatory session in the fifth year of the medical curriculum, as part of a course on *teamwork and prevention*, in Rotterdam, the Netherlands. This course consists of (1) an e-module on important teamwork principles; (2) the *Team Up*! game, where these principles can be applied with web-based patients; and (3) a (live) scenario training in a group of 12 students. The course is offered 5 times in an academic year to cohorts of approximately 60 to 90 medical students.

Before the start of the game session, all students received an email from their teacher with information about the course, study, and how to download the game on a mobile device. In the week before the study, participants were asked to read the e-module on teamwork principles at home. At the beginning of the study, students were asked to participate and (if so) to sign an informed consent form and provide permission to use their data for research purposes. All students who were invited (cohort 1: 60/144, 41.7% students and cohort 2: 84/144, 58.3% students) participated in the study (100% response), resulting in a final sample size of 144 students. Given that the study was conducted in a real educational setting, our sample size was limited to students from these cohorts.

We defined a priori that participants would be excluded when data were missing (eg, owing to technical problems) or if instructions were not adhered to. These exclusions were independent of the game scores. The main researcher was unfamiliar with the students and unaware of the group’s characteristics. Participants were divided into groups of 4 by their teacher, resulting in 36 groups of participants. During the 90-minute session, participants played the game *Team Up!* on their mobile devices. The teacher indicated which scenario they should play, consistent with the current subject matter (students within a cohort played the same scenario). All conversations in the chats were stored, including data on the scenarios and team roles. In addition, game performance data (outcomes at the 3 points in the scenario) were collected, and the time spent on the game and the team chat was recorded. Owing to practical (COVID-19–related) reasons, the first cohort played the game at home and the second cohort played it in the classroom. As students in both settings were asked to play the game as a preparation for face-to-face training and used chat for communication, we did not expect or notice any differences in responses between these 2 settings.

#### Study 2

Similar to study 1, potential participants from 2 medical centers received an email from their training coordinator, with information about the study and an invitation to participate. When they showed interest to participate, a suitable session was planned and participants received instructions on how to download the game. We were satisfied with a small group of participants compared with study 1, as this study was used as a conceptual replication study. In total, 24 health professionals (*experts*) were invited. All participants provided permission to use their data for research purposes and signed an informed consent form at the beginning of the study.

Of the 24 participants, 4 (17%) were CRM trainers from a Dutch Medical University Center and 20 (83%) were anesthesiology residents from 2 Dutch Medical University Centers. The CRM trainers participated in their free time, and the residents participated during one of their mandatory courses. Participants were divided into groups of 4 by the training coordinator, resulting in 6 groups of participants. The researcher was unfamiliar with the residents and unaware of the group’s characteristics.

Owing to practical reasons, 4 groups played the game via the web, from home, or at their workplace. Hence, 2 groups played it in a classroom. During the 90-minute session, participants played the game on a mobile device. The main researcher and coordinator were present and indicated the scenario they should play. The nature of the study was explained to the participants later.

### Materials

#### Preparatory e-Module on Teamwork Principles

In the week before the study, participants of study 1 were asked to read the e-module on teamwork principles to develop their knowledge in this field. This e-module was not mandatory and had no consequences for participants. The e-module covered the basic principles of CRM and teamwork tools and included interactive questions and patient cases to test and apply knowledge. CRM principles that were discussed include the main risks in interprofessional teamwork, situational awareness, stress management, and decision-making processes. Participants in study 2 did not have to read this e-module, as they were already familiar with these theoretical principles.

#### Multiplayer Serious Game Team Up!

*Team Up!* is designed and produced on behalf of the Erasmus University Medical Center, in collaboration with a game company, (&Ranj), to train students’ teamwork skills. It is a multiplayer game, in which 4 players have to collaborate in different roles in realistic web-simulated emergency situations.

**Figure 1:**
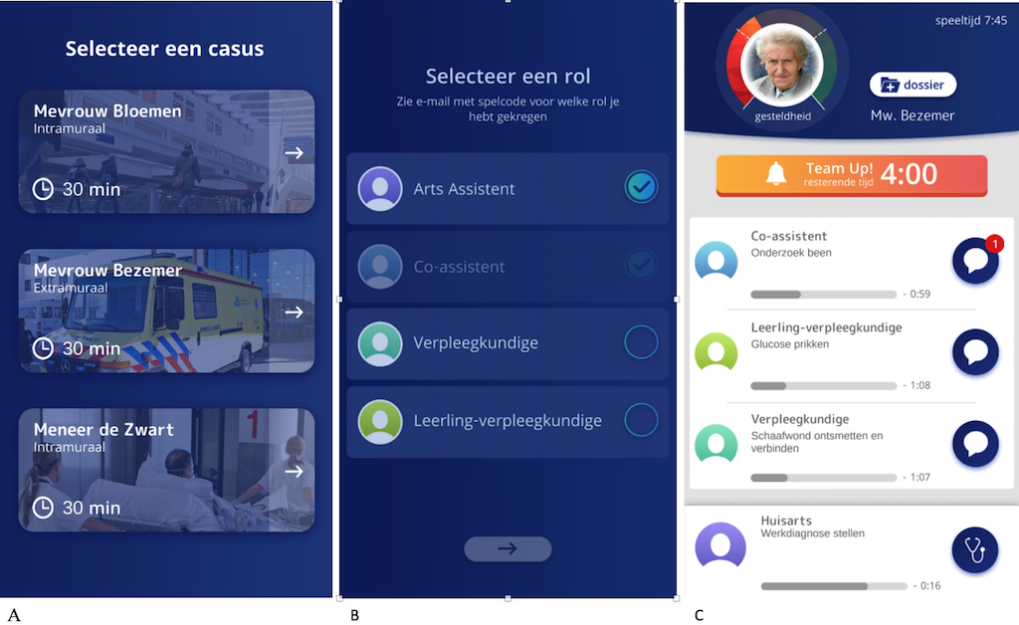
Screenshots of the game Team Up! (A) selection of patient cases, (B) selection of player's role, and (C) dashboard with the player's task progress.

Figure 1 shows screenshots of the game *Team Up!* (in Dutch). The screenshots show the screen in which players select one of the patient cases (Figure 1A); the screen in which players select a role (student nurse, intern, nurse, or physician; Figure 1B); and the player dashboard, showing (top to bottom) the patient’s condition, the remaining *team up* (group chat) time, and an overview of actions that can be performed by each role at that moment (Figure 1C).

The scenarios were developed and validated by an expert group, consisting of a clinical expert, a CRM expert, game designers, and an educational advisor. The game was designed by an experienced Dutch serious games design company and pilot-tested with the target group (medical students) before implementation. The primary aim of the game was to train students’ interprofessional teamwork skills under time pressure, using clinical scenarios. The 2 scenarios that were played by participants from this study (described in Multimedia Appendix 1) were situated in an intramural care context (patient at the hospital) and in an extramural context (patient at home).

The game contains a tutorial scenario to familiarize players with the interface of the game: one-to-one chat, group chat, actions to choose from (eg, examine the patient, determine glucose, talk to the patient’s daughter, and choose a diagnosis), overview of the patient’s general condition, and patient’s detailed file. At the beginning of the game, players select one of the 4 roles (physician, nurse, medical student, and student nurse) and can choose from several specific actions (matching their role) to provide the best possible care for a patient in a challenging and realistic medical scenario. To illustrate, in the extramural scenario, with a primary care sociomedical focus, players visit an older patient at home who has fallen and was unable to get up. They have to make a diagnosis (hypoglycemia resulting from an inadequate diet owing to forgetfulness) and provide solutions for the middle to long term (diabetes regulation, organizing meals on wheels, etc).

To collect relevant information and make correct decisions, players have to communicate effectively with each other using one-on-one and team chat functions. Individual players receive important information that they have to share in time. For instance, the student nurse talks to the daughter and hears about her forgetfulness in taking meals, and the resident receives information on the glucose level. If they fail to communicate this information effectively and make incorrect (or no) decisions, the patient’s condition will deteriorate.

Each scenario should be solved within 30 minutes. During that time, the patient’s condition is shown as improving or deteriorating (with a circle in green or red). The use of the group chat is limited in time to create time pressure. On their dashboard, players can see how much time is left for the group chat, how much time they have spent on the scenario so far, what actions they and other team members are performing, how much time this takes, the patient’s condition, buttons to the patient’s file, the team chat, and the one-on-one chats (refer to Figure 1). Each scenario consists of 3 parts. After each part, players receive brief feedback on their team’s performance—whether they completed that part of the scenario successfully, how much time it took them, and how much time was left for the team chat. For simulation training, debriefing is known to improve performance in a clinical setting [46]. Therefore, debriefing was also implemented in the game.

At the end of each scenario, players are invited to reflect on their teamwork, guided by instructions such as the following: “Have a group chat conversation on the following statement: As a team, we have collaborated and communicated effectively and efficiently.” This is followed by individual reflection, in which participants have to reflect on their own contribution to teamwork, guided by the following instruction: “Reflect on your own role, based on the next questions: What went well? What could be improved or done differently?”

The serious game, *Team Up!* is currently implemented in the (medical) curriculum of participants of study 1 (ie, a Dutch Medical University Center). The game is used as web-based preparation for face-to-face scenario-based interprofessional teamwork training, followed by individual assessment of teamwork skills.

### Data Analysis

We conducted qualitative analyses, using a deductive approach, on the conversations in the chats. We started with concepts attributed to teamwork, more specifically CRM principles [19], and tested its implications in our chat data. For study 1 (among students), the first author (LP) and second author (TF) developed an initial coding scheme. Thereafter, they independently read and coded the conversations of 1 group in the first cohort line by line to capture concepts related to CRM. After a discussion between the raters about the discrepancies until consensus was reached, the initial coding scheme was refined to obtain a conceptual framework for chat conversation analyses (Multimedia Appendix 2). The remaining conversations of the groups in the first cohort were coded by LP. Next, LP and a research assistant (Roan Kasanmonadi) independently read and coded the conversations of 1 group in the second cohort. After the discrepancies were resolved by discussion, Roan Kasanmonadi coded the remaining conversations of the groups in the second cohort. Points for discussion were constantly discussed with LP and TF. The research team subsequently categorized the codes and identified the relationships between the CRM themes. During the final stage of analysis, the research team discussed the CRM themes and the extent to which the CRM principles were used in the game. To facilitate this process, LP constructed concept maps, presenting main themes, subthemes, and their relationships. LP, TF, and Roan Kasanmonadi captured reflections, ideas, and interpretations in memos throughout data analysis. Data analysis was supported by the qualitative data analysis and research software ATLAS.ti [47].

The qualitative analyses for study 2 (among experts) were conducted in a similar manner as in study 1. However, this time, the first author (LP) and a research assistant (Joost Jan Pannebakker) independently read and coded the conversations of 1 group line by line to capture concepts related to CRM. After a discussion about the discrepancies until consensus was reached, the remaining conversations of the groups were coded by Joost Jan Pannebakker. The research team subsequently discussed the CRM themes.

## Results

### Overview

The results of our analyses are described according to a conceptual framework of important teamwork principles in five main themes: (1) creating shared situational awareness, (2) decision-making, (3) communication, (4) team management aspects, and (5) team debriefing. Refer to Multimedia Appendix 2 for the coding scheme.

We will describe the results of study 1 (among medical students) and study 2 (among experts) combined, as the findings may be group-related (students are novices in teamwork) and it seems interesting to compare the findings between groups with different experiences.

### Shared Situational Awareness

In the team chats in study 1, we observed elements of trying to build and maintain shared situational awareness. Participants sometimes tried to create a comprehensive overview of the patient’s situation by asking for missing information or by summarizing, using the team chat function. The created situation overview usually remained fairly general; requesting additional views or information was often lacking. Textbox 1 shows examples of aiming for shared situational awareness.

In study 2, similar to study 1, participants tried to create a comprehensive picture of the situation by asking for missing information or by summarizing. They often summarized efficiently (ie, point by point) and quickly returned to their own tasks. The created picture of the situation (again) usually remained fairly general; follow-up from the other team members was often lacking. Textbox 2 shows an example of the chat.

**Shared situational awareness—examples from 2 student groups.**

**Example 1**

- “Sorry, did anyone ask about her eating pattern? Or her compliance to therapy? We need to know those two things. Yes, she finishes her plate ha-ha. Don’t know about therapy compliance. She takes all of her medication. Okay, I know enough now.” [Group 4; cohort 1]

**Example 2**

- “What information do we have right now? Glucose is 2,8. Fell down, she did not feel that coming, does not know whether she’s been unconscious. Is feeling sweaty now. No further complaints. No chest pain, no heart palpitations. No loss of feeling in arms or legs. That’s it, I believe. Great. No wounds? No. Intox? Do you know whether she fell on her head?” [Group 23; cohort 2]

**Shared situational awareness—example from an expert group.**

**Example**

- “We think UTI [urinary tract infection] with SIRS [systematic inflammatory response syndrome] and Hypoglycemia right? Yes agree. Yes she’s confused, but no further neurological abnormalities.” [Group 5]

### Decision-making

In study 1, among students, we observed decision-making aspects such as re-evaluating decisions or situations and anticipating and planning (Textbox 3 shows a few examples).

In almost all groups, participants regularly set priorities and focused on issues that needed attention first. Usually, this happened when a team member identified that the patient’s condition was deteriorating. Participants either indicated that something needed more attention or asked their team members what should be done first. In most cases, this was followed up by the team members; they subsequently shared and requested information or shared their own thoughts. This is consistent with the objective of allocating attention wisely, that is, looking at the overall picture of the situation at set times. Remarkably, a justification for why something should be prioritized was often lacking. Therefore, the team members were less involved in a joint thinking process and were rarely asked about their motives.

In study 2, among experts, we observed a wide range of decision-making processes: re-evaluating the patient’s progress, noticing changes in the patient’s condition, and subsequently, making judgments about continued care or treatment adjustments (refer to Textbox 4 for examples). Re-evaluations of decisions or judgments seemed to be more present in the experts’ chats compared with students’ chats. Similar to participants of study 1, they did not use explicit prevention of fixation errors.

In almost all expert groups, players—again—regularly set priorities and focused on issues that need attention first. Usually, they either indicated that something needed more attention or asked their team members what should be done first. In most cases, this was followed up by the team members; they subsequently shared and requested information or shared their thoughts. Compared with students, experts more often seemed to justify why something should be prioritized.

**Decision-making—examples from 3 student groups.**

**Example 1**

- “We have to check whether she has a fall spot on her eye or head.” [Group 16; cohort 2]

**Example 2**

- “What room are we going to? Wat type of patient is coming? Do we all need to go to one room? We all need to go to the same room. This can be done in a normal room. I think acute. Can possibly deteriorate right? I would choose acute. Acute room? Okay.” [Group 19; cohort 1]

**Example 3**

- “First correct her Hypoglycemia, then stabilize, then examine leg.” [Group 21; cohort 2]

**Decision-making (re-evaluation)—examples from 2 expert groups.**

**Example 1**

- “Still sounds as a hypo.. delirium..” [Group 7]

**Example 2**

- “Continue with the present plan?” [Group 7]

### Communication

In study 1, our analyses showed that general communication techniques, such as sharing and requesting information, were frequently used. More specific communication techniques such as confirming a request (*closed loop*) and inviting other team members to add ideas or thoughts (*speak up*) were also observed, both in the team chat and one-on-one chats (Textbox 5).

Regarding *closed loop communication*, the recipient usually confirmed the request (refer to examples in Textbox 5), but the confirmation by the requester that this message has arrived was usually lacking (although this is not strictly necessary in closed loops). In addition, the completion of a task was not always confirmed to the requester in the chats. This may be caused by the game layout, in which the completed task remained visible.

Regarding *speak up*, results showed that participants used it quite often. Inquiries were regularly initiated. Furthermore, thoughts and hypotheses were shared and subsequently discussed; however, they were quite brief discussions with few open questions.

Taking a time-out was not often explicitly mentioned in the chats. Time-outs were mainly used to bring focus and coordinate with each other. Starting the team chat in the game may also be considered as taking a time-out. Participants were informed before playing the game that they could use the team chat to coordinate with each other or to check whether they all agree. The team chat was mainly used to share and request information among all team members.

In study 2, among experts, general communication techniques such as sharing and requesting information were also often used. Participants communicated very succinctly and efficiently, which is probably the result of their experience in the clinical workplace. Compared with students, they communicated relatively more according to the *closed loop* principle—repeating that a requested task will be performed. Textbox 6 shows a few examples.

Requests were usually repeated by the recipient, but the requester seldom acknowledged that this message had arrived. Moreover, the completion of a request was generally not confirmed to the requester.

*Speak up* was used relatively more often by participants in study 2 (experts) compared with participants in study 1 (medical students). When one of the team members discovered a discrepancy or vital new information or when they had doubts about something, they often shared it with the other team members and acted upon it. Although thoughts and hypotheses were again often shared, the expert participants more often used open-ended questions to collect additional information from the other team members.

Explicitly taking a time-out to bring focus and to coordinate with each other was only observed once in the chat data. However, as stated previously, one could argue that starting the team chat implies taking a time-out. Participants used the team chat effectively by regularly summarizing the available information, discussing the status quo, and ensuring agreement. Usually, 1 team member summarized, whereas ideally, they would also complement each other.

**Communication (closed loop and speak up)—examples from 3 student groups.**

**Example 1**

- “Could you inform the daughter? Yes I will.” [Group 4; cohort 1]

**Example 2**

- “Can you do the urine test? Yes, I’ll do the urine test.” [Group 8; cohort 2]

**Example 3**

- “Everyone in agreement? Yes. Yes. Yes.” [Group 13; cohort 2]

**Communication (closed loop and speak up)—examples from 3 expert groups.**

**Example 1**

- “Shall I give her a coke?

Yes give her a coke.

I’ll give a glass of coke to the patient.” [Group 3]

**Example 2**

- “What are we thinking of?” [Group 4]

**Example 3**

- “I have the feeling we are missing something” [Group 7]

### Team Management

We observed some team management principles in study 1, such as appointing leaders and followers, distributing the workload, and calling for help. Not all teams appointed leaders. Cognitive aids were rarely used. The division of team roles and workload was sometimes discussed at the beginning of the gameplay. When it was decided or mentioned who would take the lead, the leader immediately assumed that role. They distributed the workload among team members—which was rarely observed in groups without a clear leader—and checked whether all team members agreed to a major decision. In these groups, teamwork improved; the team chat was used functionally, and team members communicated effectively, for instance, by using closed-loop communication. The division of roles was discussed more often during the group debriefing (refer to the following paragraph), that is, *after* the scenario had been completed.

In contrast to our findings in study 1, the division of roles—including designating leaders and followers was frequently observed in the chat data of study 2. When participants assigned a leader, that leader frequently distributed the workload among team members, took the initiative in determining which working method is to be followed, and assumed responsibility for overseeing the situation (refer to Multimedia Appendix 3 for examples). Again, management principles, such as calling for help and using cognitive aids, were rarely observed in this group. Examples of team management principles of both groups are presented in Multimedia Appendix 3. Designating leaders and followers appears to be related to effective communication between team members, as teamwork principles were better applied in groups in which roles were clearly divided. Again, the division of roles was discussed more often during the group debriefing, *after* the scenario had been completed.

### Team Debriefing

Analyses of the debriefing chats in study 1 (students) revealed that participants were usually able to reflect on their teamwork and identify the causes of both good and poor teamwork. This sometimes led to agreements regarding better division of roles in the next scenario (refer to Multimedia Appendix 3 for an example). In addition, some groups also discussed their communication and how to improve this in the subsequent scenario. However, in general, the groups only identified what went well and what went wrong in their communication and did not explicitly discuss the follow-up steps.

Results from study 2 (experts) showed that participants were usually able to identify the causes of both good and poor teamwork during the team debriefing (refer to Multimedia Appendix 3 for examples). However, this rarely led to concrete agreements for the next scenario. Discussions about communication were also limited; that is, they mainly briefly identified what went well and what went wrong in terms of communication and did not explicitly discuss the follow-up steps.

Sometimes, team members took the initiative to start a discussion with the entire team about their teamwork and communication, but there was rarely any response. Examples of debriefing by both groups are presented in Multimedia Appendix 3).

### Summary of Results

In summary, our findings showed that both students and experts used important teamwork principles during gameplay, such as creating shared situational awareness, decision-making, general and specific communication strategies (eg, closed loops and speak up), team management actions (dividing tasks), and debriefing reflections. Experts also used some principles that students rarely used (eg, appointing leaders and dividing tasks). Experts used more open communication strategies (eg, speak up), performed more re-evaluation, and provided better justification of decisions. Prevention of fixation errors and use of cognitive aids were less observed among both groups, and debriefing conversations in both groups remained relatively superficial.

## Discussion

### Principal Findings

In this study, we examined the teamwork principles that were applied in a multiplayer serious game by medical students and teamwork experts. We found that both undergraduate students and experts practiced important teamwork principles during gameplay, such as shared situational awareness, decision-making (re-evaluation and prioritizing), communication (closed loop and speak up), and team management (appointing a leader and distributing the workload). Among experts, we observed the use of similar teamwork principles often on a moderately high level.

The teamwork game was designed to allow medical students to practice teamwork principles during medical emergencies. Findings can contribute to a better understanding of the potential of multiplayer serious games for developing these skills and can inform designers of blended teamwork training. We conducted qualitative analyses, using a deductive approach, on the conversations in the chats. We started with a conceptual framework of important teamwork themes (a CRM framework [19]): creating shared situational awareness, decision-making, communication, team management, and debriefing. We conducted 2 qualitative studies: a main study among medical students and, as a conceptual replication study, a study among teamwork experts (experienced residents and trainers in teamwork).

Our results showed that both students and teamwork experts applied most teamwork principles (ie, the CRM principles) during gameplay. Among students, we observed the creation of shared situational awareness, decision-making, important communication, and team management principles (closed loop and distributing the workload). Some teamwork principles were less or rarely used, such as requesting additional views when creating shared situational awareness, preventing fixation errors, appointing leaders, and applying speak up with open questions. Debriefing was usually conducted superficially—a brief exchange of good and poor teamwork elements, without a thorough discussion about the team members’ contributions and how it may be improved in the next scenario. In general, the more complex elements of teamwork were less observed in the web-based game played by the students. Among experts, we also observed the use of all main teamwork principles. However, they used more re-evaluation, closed-loop communication, and open questions with speak up, and they appointed leaders and divided roles more often than the students. In general, compared with the students, the experts’ communication was more efficient and succinct, and they substantiated the assumptions more often, matching their expertise level.

The fact that most teamwork principles were applied in this multiplayer game indicates that games can provide a flexible and activating learning environment in which teamwork principles can be exercised safely on a basic level. Considering the importance and complexity of these skills and the fact that face-to-face training is cost intensive, this is a promising finding for educational practice. Students can use these scenarios as often as they want, in different roles, and with different peers. Once developed, this type of game can be frequently used for training, without extra costs. Moreover, for students, this type of game provides the opportunity to apply and *experience* teamwork principles in a realistic context, which is much more meaningful and effective than reading about these principles [48].

In the current (*Team Up*!) game design, the appearance of specific role-related tasks and information, in combination with the one-to-one and team chats, created the necessity and opportunity to collaborate. The web-based patient, deteriorating or improving during gameplay, probably created a sense of urgency and feedback. Previous study has shown a consistent association of technology-enhanced simulation training in health professions education with positive effects on knowledge, skills, and behaviors [35]. However, this is not yet established for serious games specifically and, therefore, more validation of this format is needed [44]. Furthermore, more studies on the effectiveness of specific design choices in games are needed [49], to know what characteristics facilitate the use of communication and teamwork.

The observation that teamwork experts applied some principles on a more elaborate level (eg, more open questions and more closed-loop communication) may indicate that the use of these principles is related to expertise. The game scenarios facilitated the use of these principles for teamwork experts on a higher level than for novices. Hence, the game appears to be a promising learning environment for training these skills for groups with different experiences. Games have been shown to aid the development of complex skills in individual medical practitioners [40]. In this study, we demonstrated that a multiplayer game could elicit the application of teamwork principles both in novices and experts. As the application of these principles is essential to learn the skills, we view this study as a vital first step in investigating the potential of multiplayer game–based learning for teamwork skills. It would be interesting to investigate what support novices need to apply these skills on a more elaborate level through further studies. Examples of potentially helpful support are checklists (eg, with CRM principles) in the game. Checklists can act as task support that helps students adhere to certain principles or procedures [37].

For simulation training, debriefing is known to improve future performance in a clinical setting [46], and therefore, it is a crucial part of the training. In this study, we observed that debriefing as a group (after performing the web-based scenarios) was conducted superficially by both students and experts. The general questions that were provided during debriefing in the game led to a superficial discussion on teamwork and roles. Given the relatively unguided format, it may not be surprising that most player groups did not engage in deep reflection. In general, reflecting in depth as a group is difficult without instructor-led guidance or a more structured format; however, some groups managed to reach a deep analysis level. According to the Promoting Excellence and Reflective Learning in Simulation model of debriefing, including reaction, description, analysis, and application [50], most participants were in the reaction phase. This raises the question of how the transition to the description or analysis phases can be stimulated. Using instructor-led guidance is not a solution in a game context, as this would severely limit the possibilities of flexible learning and entail high costs. Different studies have showed that structured self-debriefing can be equally effective as instructor-led debriefing [51,52]. Furthermore, Van der Meij et al [53] suggested describing specific and observable actions and criteria for good teamwork in the debriefing format. Further studies could clarify the effectiveness of this type of debriefing formats in improving the quality of reflections and team debriefing.

Some specific teamwork principles were not applied by both player groups, such as using cognitive aids and preventing fixation errors. This is likely to be related to the scenarios (these teamwork principles may not be needed for solving specific patient scenarios) or the context of the game (owing to functionality or interface). Some game functions appeared to limit the possibilities to use specific teamwork principles. To illustrate, it is probably not logical to apply closed-loop communication when the messages remain in the chat and when the tasks that team members have performed remain visible in the game app (as opposed to verbal instructions in the real-life environment). In face-to-face training, these less-used principles can be given extra attention. We also noticed that some principles, such as planning and decision-making (eg, while preparing for the hospitalization of a patient), were observed more often in some scenarios than in others. This implies that offering a variety of game scenarios, aligned with specific teamwork learning goals, is important. In addition, some more complex teamwork principles can probably best be trained in a face-to-face simulation setting. Then, the multiplayer game can serve as an (efficient) preparation for skills training in a blended design.

### Strengths and Limitations

In this study, we investigated whether a multiplayer serious game, used in a medical curriculum for fifth-year medical students in the Netherlands, facilitates the use of important teamwork principles. We were able to confirm that all main principles were applied in the web-based patient scenarios. As described previously, these results have important educational value for training students in teamwork skills.

A limitation of this study is that we did not investigate whether the gameplay actually improved participants’ teamwork skills or whether transfer of teamwork skills to a setting outside the game occurred. Instead, we deliberately chose to investigate the teamwork principles that were used during gameplay because the game was implemented in the skills training in our curriculum and we did not want to deny students access to the game (as would be necessary for a randomized design with a control group). This would be an interesting object for future studies, for example, comparing a group that used the teamwork game with a group that watched a video on teamwork principles, followed by scenario assessments.

Another limitation is the difference in recruitment between the groups of participants of study 1 (students) and study 2 (experts). Regarding students, the whole semester group played the game (as part of their curriculum) and was approached for the study. Regarding experts, specific groups of experts were asked to play the game and participate in the study (outside the context of a course). We believe that this was inevitable because the game was part of the medical curriculum and acceptable because the expert data were merely used as an additional benchmark. Although this made a direct comparison between the 2 groups more difficult, we did not see indications of biased results.

### Conclusions

In this study, we examined the teamwork principles that were applied in a multiplayer serious game by medical students and teamwork experts. We found that the game facilitates the application of important teamwork principles among medical students, and they were related to all main teamwork themes: shared situational awareness, decision-making (re-evaluating and prioritizing), communication (closed loop and speak up), and team management (appointing a leader and distributing the workload). Among teamwork experts, we observed the use of similar teamwork principles often on a higher level: more justification of decisions, re-evaluation, closed loops, and open questions.

A multiplayer game to train teamwork skills appears to be a promising learning environment, as it can be used as a flexible training tool to safely practice teamwork principles and prepare for face-to-face training. Hence, during face-to-face training, students can focus more on exercising complex teamwork principles and processing feedback.

## Appendix

Multimedia Appendix 1 Description of scenarios 1 and 2.

Multimedia Appendix 2 Coding scheme for the teamwork principles.

Multimedia Appendix 3 Textboxes with examples from chats on team management and debriefing.

## Abbreviations

CRM: Crew Resource Management
