# Supplementary material for: Teamwork Training With a Multiplayer Game in Health Care: Content Analysis of the Teamwork Principles Applied
Source: JMIR Serious Games. 2022 Dec 9;10(4):e38009. doi: 10.2196/38009 (PMC9789497; doi:10.2196/38009)
Supplement: Multimedia Appendix 1 [file games_v10i4e38009_app1.docx]

## Appendix A.

# Description of Scenarios 1 and 2

**Scenario 1: Mrs. Bloemen** **(Intramural scenario)**

Friday 4.00 PM, a 58-year-old woman presents at the ER with fever, abdominal pain and confusion. She has a history of type 1 diabetes mellitus and renal failure, for which she receives hemodialysis three times a week.

Diagnosis: Sepsis caused by a urinary tract infection: Urosepsis.

The players take on the role of resident, medical intern, nurse and student nurse.

**Scenario 2: Mrs. Bezemer (extramural scenario).**

Wednesday morning at 11.00 AM, you all arrived at Mrs Bezemer’s house. Fifteen minutes ago she called you (using her alarm) because she had fallen and was unable to get up.

Diagnosis: Hypoglycemia caused by a poor diet as a result of forgetfulness, possibly aggravated by emotional problems.
Players take the role of a family physician, medical intern, nurse and student nurse.
