# Supplementary material for: Teamwork Training With a Multiplayer Game in Health Care: Content Analysis of the Teamwork Principles Applied
Source: JMIR Serious Games. 2022 Dec 9;10(4):e38009. doi: 10.2196/38009 (PMC9789497; doi:10.2196/38009)
Supplement: Multimedia Appendix 2 [file games_v10i4e38009_app2.docx]

## Appendix B

# CODINGS SCHEME TEAMWORK PRINCIPLES

Operationalization of teamwork concepts in a coding scheme for the game chats. Teamwork concepts in bold are the main concepts; non-bold printed concepts are parts of the main concepts.

| Concept | Description | |
| --- | --- | --- |
| Shared situational awareness | |  |
| Situational awareness | By using clear and unambiguous communication, all team members share a common notion of the (urgency of the) actual situation. |  |
| Decision-making | |  |
| Focus your attention | **Focus on problems that are most urgent or alternately focus on details and the big picture. A goal can be to reconsider the overall situation now and then.** |  |
| Prioritize | **Prioritize. The treatment of a patient’s vital problems, for instance, should always come first.** |  |
| Re-evaluate | **Re-evaluate, for instance regarding decisions that are previously taken or on the critical parameters**. |  |
| Prevent fixation errors | **Prevention of ‘fixation errors’ (incorrect interpretation of a situation) can be obtained by creating a ‘fresh look’ at the situation: asking for a second opinion from a colleague and trying to leave out earlier assumptions. For example, inviting team members to formulate hypotheses or critique a hypothesis or applying the ‘10 seconds for 10 minutes rule’**. |  |
| Anticipate and plan | **Going through a number of steps before starting a procedure. Anticipate problems that might be expected, prepare measures for expected barriers.**  **Know the work environment: who can be called when help is needed? How can this person be reached most quickly? How can help arrive in place? Where is the equipment needed and how does it work?** |  |
| Communication | |  |
| Share and request information | Sharing and requesting information. |  |
| Informing on actions | Informing team members on actions to be executed or finished actions. |  |
| Informing on results | Informing team members on test results or certain knowledge/ protocols. |  |
| Request for actions | Requesting a team member to perform a certain action or whether it has been performed. |  |
| Closed-loop communication | When team members get a request or assignment to perform an action, they repeat this request. Ideally, s/he waits until the requester confirms that the assignment is correctly repeated, but this is not a necessity. Let the requester know that the assignment is performed and wait until this is confirmed. |  |
| Speak up | Speak up. Ask team members whether they would like to add something or whether they have any thoughts. |  |
| Cross (double) check | Cross-check to verify certain findings by using different resources. For example, double-check the patient’s identity by checking the wristband, asking for the patient’s name and date of birth or checking the file. And performing a joint check of medication before administering. |  |
| Take a timeout | Taking a timeout |  |
| Summarize | Summarizing the current situation (creating an overall picture) to create a joint vision of the patient's situation. The goal may be to prevent fixation errors or to create situational awareness. |  |
| Team management aspects | |  |
| Appoint leaders and followers | It is important, at all moments and for every team member, that it is clear who is the team leader. The leader keeps an overview, communicates clearly with team members, and divides the workload. The leader is not necessarily the highest person in the hierarchy. Equally important is followership: executing assignments as well as possible and thinking along. |  |
| Distribute the workload | Distributing the workload |  |
| Call for help | Recognizing one’s limits and asking for help in time. For example, consulting with a supervisor, calling an emergency team, or consulting a colleague. |  |
| Use cognitive aids | Retrieving information and using tools such as checklists, protocols, and a calculator. Calling helplines such as the National Poisons Information Center. |  |
| Team debriefing |  |  |
| Reflection on teamwork | Reflection on teamwork during the team debriefing. |  |
| Reflection on communication | Reflection on team communication during the team debriefing. |  |
| Invitation to participate or add | Team members are invited to participate and add their reflections and conclusions during the team debriefing. |  |
