# Supplementary material for: Teamwork Training With a Multiplayer Game in Health Care: Content Analysis of the Teamwork Principles Applied
Source: JMIR Serious Games. 2022 Dec 9;10(4):e38009. doi: 10.2196/38009 (PMC9789497; doi:10.2196/38009)
Supplement: Multimedia Appendix 3 [file games_v10i4e38009_app3.docx]

**Appendix 3: Textboxes with examples from chats on Team management and Debriefing**

Team management; examples from 2 student groups

| *Example 1*  *“Hi, I’ll divide some tasks! Check your individual chats.”* (Group 2, cohort 2)  *Example 2*  *“Can the intern help with the physical examination? Then the nurse can take a history.”* (Group 13, cohort 2) |
| --- |

Team management; example from 1 expert group.

| *Example 1*  *“In one minute we’ll have a team meeting.”* (Group 3) |
| --- |

Debriefing; examples from 3 student groups.

| *Example 1:*  *“At the start, it was a bit chaotic. We did not divide the tasks that well yet.”* (Group 8, cohort 2)  *Example 2:*  *“A leader was missing. Do we agree that the physician keeps the overall picture? I noticed that everyone was speaking at the same time. The physician should take the lead. The physician will be the leader in the next scenario.”* (Group 1, cohort 1)  *Example 3*  *“We need to better discuss what we're doing. Because sometimes 2 or 3 people do the same thing. And maybe also pass on what information we receive. Anyway, talk to everyone before you do anything. Then it is not double. Unless it's acute*.” (Group 17, cohort 1) |
| --- |

Debriefing; examples from 3 expert groups.

| *Example 1*  *“I felt part of the team.”* (Group 5)  *Example 2*  *“How did you all think it went? We improved our tuning in after round 1.”* (Group 7)  *Example 3*  *“Was your input sufficiently used?”* (Group 5) |
| --- |
